# Supplementary material for: Study of the Antibacterial Capacity of a Biomaterial of Zeolites Saturated with Copper Ions (Cu2+) and Supported with Copper Oxide (CuO) Nanoparticles
Source: Nanomaterials (Basel). 2023 Jul 24;13(14):2140. doi: 10.3390/nano13142140 (PMC10384100; doi:10.3390/nano13142140)
Supplement: Supplementary file 1 [file nanomaterials-13-02140-s001.zip › nanomaterials-2444156-supplementary.pdf]

Supplementary Materials

Study of the Antibacterial Capacity of a Biomaterial of Zeolites Saturated with Copper Ions (Cu^{2+}) and Supported with Copper Oxide (CuO) Nanoparticles

Lina M. Romero [1], Nicolas Araya [1], Daniel A. Palacio [2], Gabriela A. Sánchez-Sanhueza [3], Eduardo G. Pérez [4], Francisco J. Solís [4], Manuel F. Meléndrez [1,5] and Carlos Medina [6,*]

* Correspondence: cmedinam@udec.cl; Tel.: +56-4-12203187

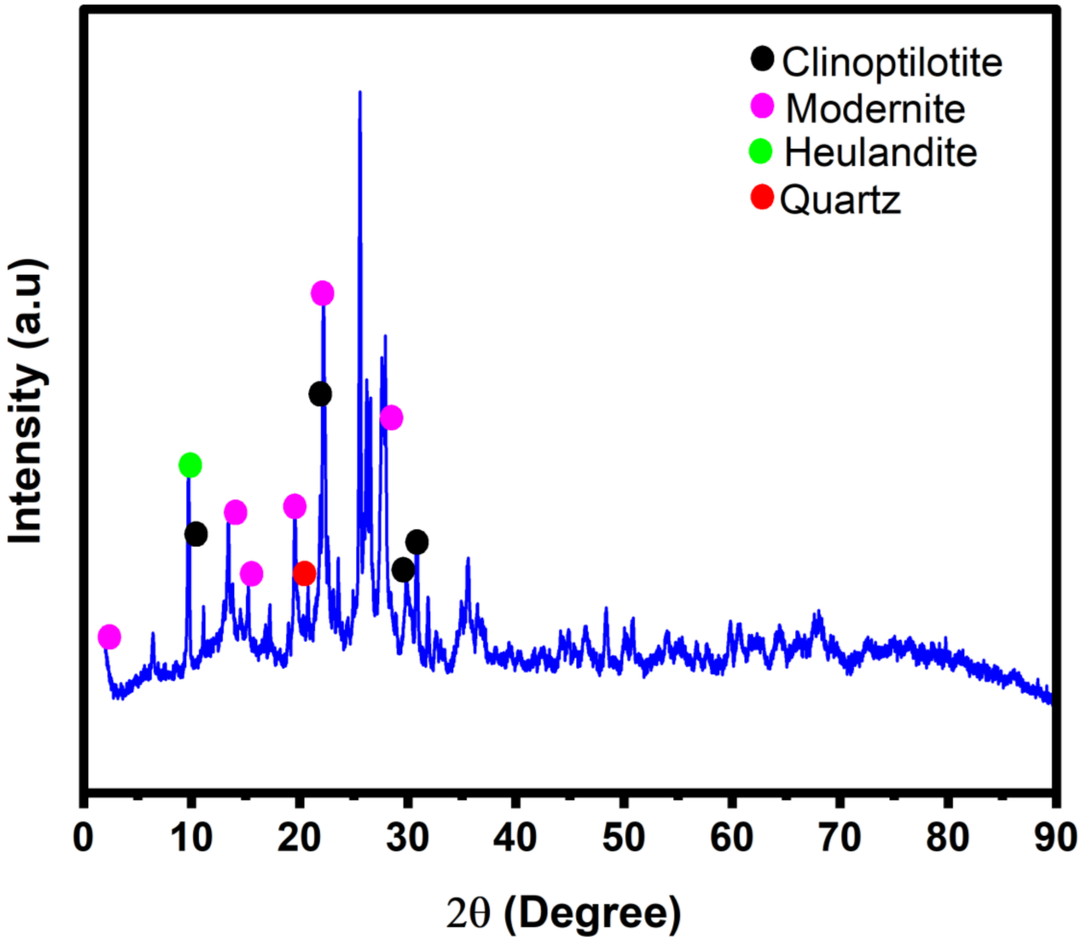

Figure S1: XRD pattern of natural zeolite (ZLn).

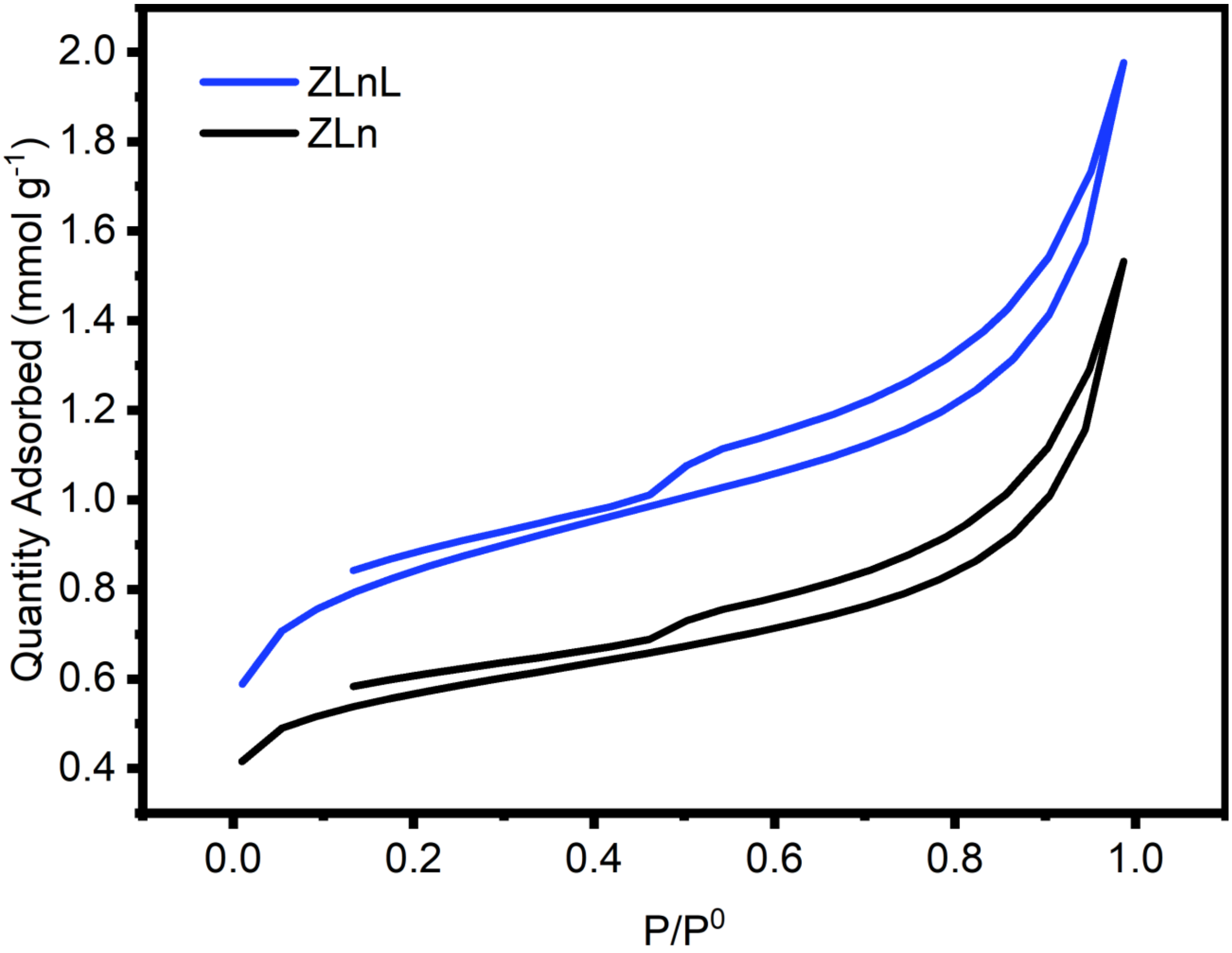

Figure S2: N_2 adsorption-desorption curve of ZLn and ZLnL.

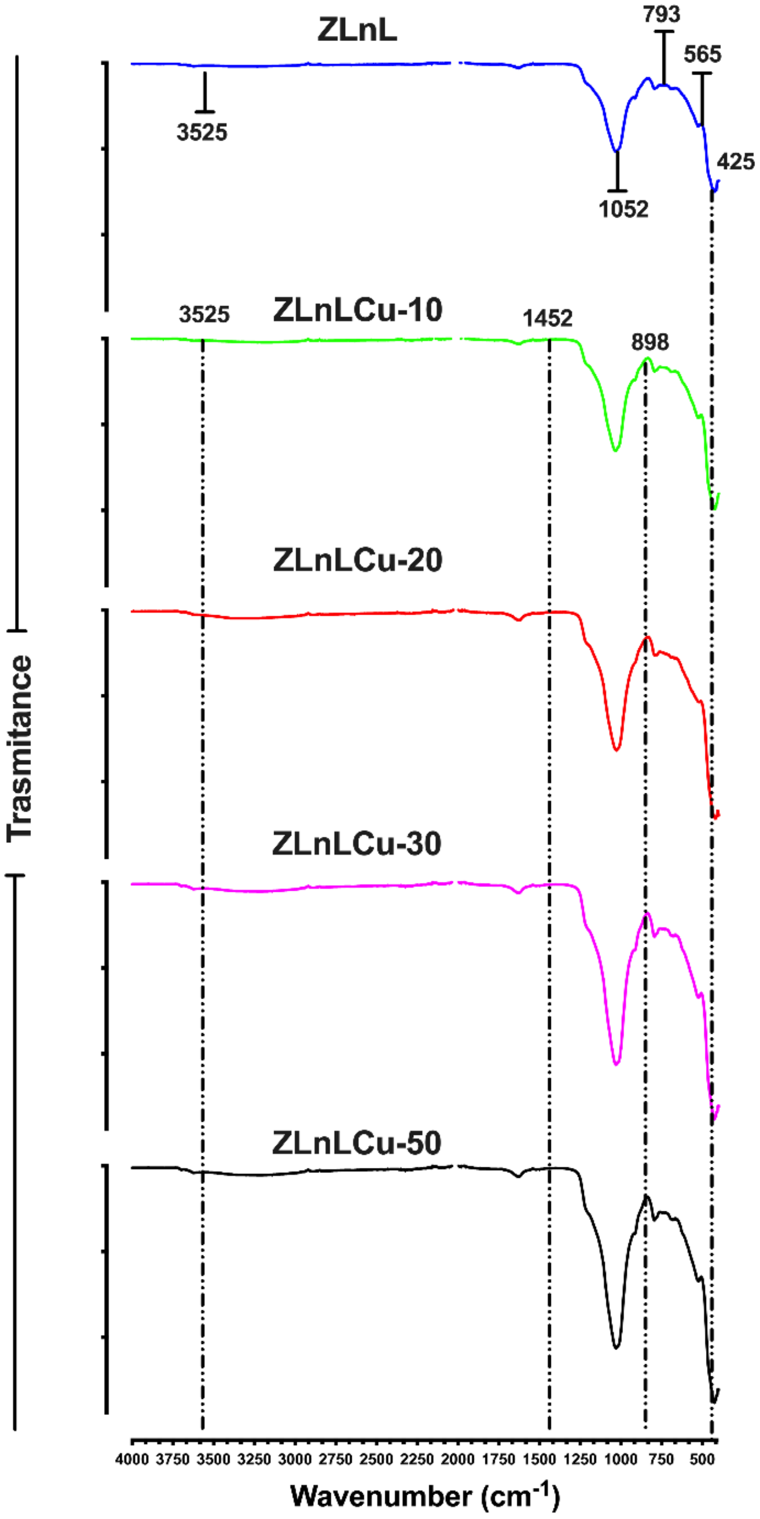

Figure S3: FTIR spectra of ZLnL before and after Cu^{2+} ion adsorption.

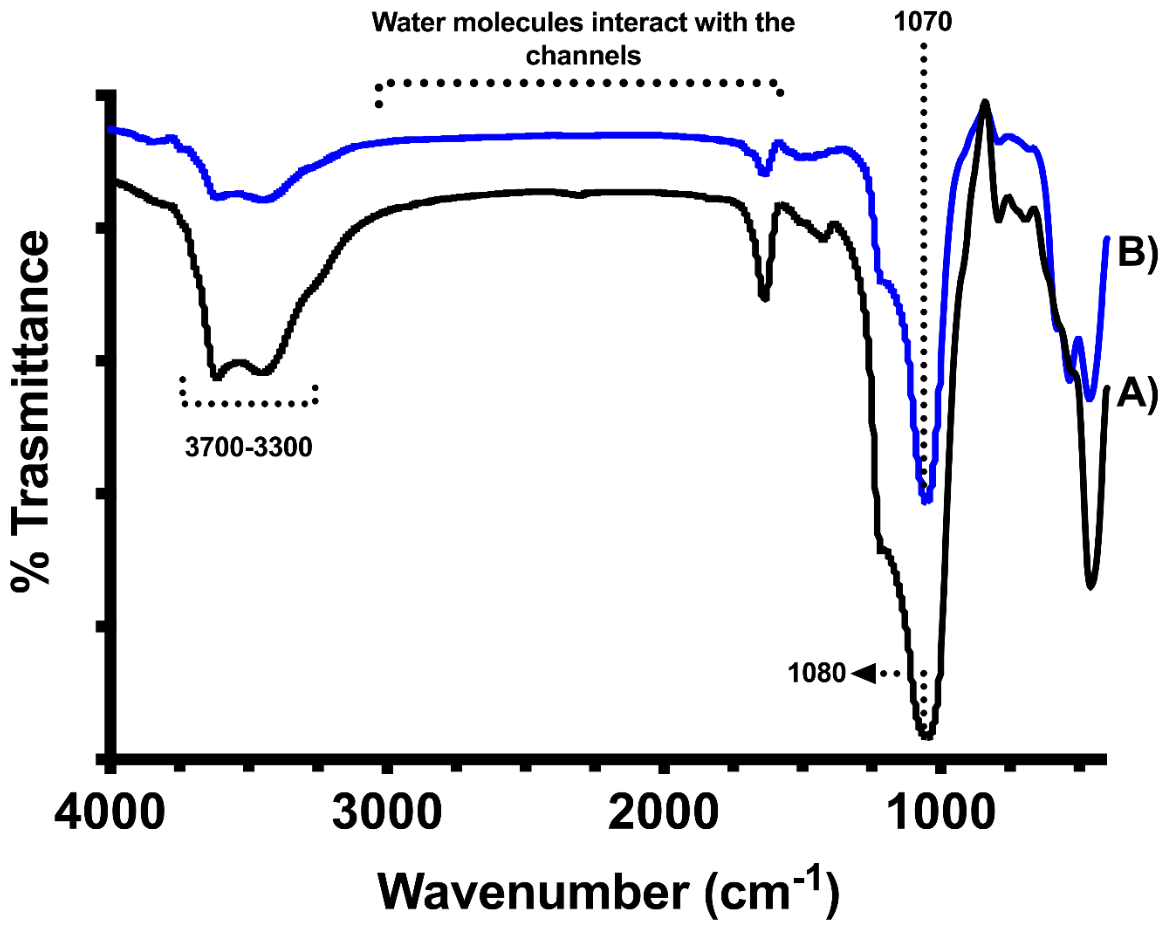

Figure S4: FTIR spectra of (A) ZLnL and (B) ZLnLCuONPS.
